# Space and time in episodic memory: Effects of linearity and directionality on memory for spatial location and temporal order in children and adults

**DOI:** 10.1371/journal.pone.0206999

**Published:** 2018-11-08

**Authors:** Thanujeni Pathman, Christine Coughlin, Simona Ghetti

**Affiliations:** 1 Department of Psychology, York University, Toronto, Ontario, Canada; 2 Department of Psychology, UC Davis, Davis, California, United States of America; 3 Center for Mind and Brain, UC Davis, Davis, California, United States of America; French National Center for Scientific Research (CNRS) & University of Lyon, FRANCE

## Abstract

Episodic memory is a critical capacity that involves remembering past events along with their spatial and temporal contexts. Relatively little is known about the relations between spatial and temporal information in long-term memory in children or adults. The present research examined the influence of the mental timeline (linear horizontal display extending from the left to right direction for English speakers) on memory for events and their spatial and temporal features in 7-year-olds, 9-year-olds, 11-year-olds and young adults (*N* = 146). During encoding, participants studied triplets of objects, varying on two dimensions of the mental timeline: linearity (whether objects were presented in linear succession or not) and direction (whether objects were presented from left-to-right or right-to-left). After a delay, during retrieval, participants were tested on their memory for individual objects, and either the spatial location or temporal order of the objects. We found that overall accuracy for spatial location was higher than accuracy for temporal order, and there was a parallel developmental trajectory for both these aspects of memory. Across age groups we found that memory for temporal order, but not spatial location, was influenced by linearity and direction (i.e., match to mental timeline). Thus, in both children and adults the spatiotemporal mental timeline supported memory for temporal order, converging with predictions generated within domains of language and thought and enhancing our understanding of how space and time are represented in the mind.

## Introduction

Episodic memory allows us to recall specific past events from a particular place and time [1, 2]. Our ability to remember an event along with its spatial context (i.e., where events occurred) and temporal context (i.e., when events occurred) is critical for daily functioning. We use it for relatively trivial tasks, like recalling where we parked our car this morning or the restaurant we went to for lunch. We also rely on episodic memory for highly consequential tasks, such as recalling whether we turned off the kitchen stove before we left our house, or where we put our medicine bottle after taking our pills. Although there has been extensive research on the binding of spatial and temporal features of events, most studies have investigated spatial and temporal memory separately, and little is known about how spatial and temporal relational mechanisms relate in long-term memory. Further, relatively little is known about the development of spatial and temporal memory. Although infants and young children are capable of remembering specific past events [3], age-related improvements in episodic memory are apparent even in middle to late childhood [4–9]. Given that memory for spatial and temporal context are defining features of episodic memory, an understanding of episodic memory development would be incomplete without an understanding of memory for spatial and temporal context in childhood. In the present investigation, we examined memory for spatial and temporal context in school-aged children and young adults.

### Spatial and temporal representations in the mind

From Aristotle and Descartes to Newton and Einstein, discussions and debates about space and time are long-standing in physics and philosophy [10]. More recently, researchers have examined the relations between space and time in the mind. In the domains of language and thought there is evidence that representations of time are more influenced by space than vice versa. In the English language, for example, it is more common to use spatial words to describe temporal information (e.g., “The appointment was *pushed back*) than to use temporal words to describe spatial information [11, 12]. Moreover, Boroditsky [13] experimentally tested the relation between space and time in language and found that priming adults to think about spatial information influenced their answers to ambiguous temporal questions, but priming adults to think about temporal information did not influence their answers to ambiguous spatial questions. In psychophysical experiments, again, researchers find that space influences time more than vice versa: when human adults and children, but not monkeys, make judgments of space (distance) and time (duration), irrelevant spatial information influences time judgments more than irrelevant temporal information influences space judgments [14–17]. Neural activations as adults make these judgments also show asymmetry between space and time [18]. Although there is controversy in the literature about the theoretical model that describes the relations between space, time and other magnitudes [16], this body of work suggests an asymmetry between space and time: temporal representations are influenced by spatial representations in the mind but not necessarily vice versa.

Why is there such an influence of space on time? Research points to the *mental timeline*: time is represented as a linear spatial display in the mind. The mental timeline is inherently spatiotemporal. It involves a physical or a mentally pictured line, but what makes it more than just a line is the fact that it requires ordered placement on that line. There is evidence that the mental timeline is in the same direction as reading in both sighted (printed text) and blind (braille) individuals [19, 20], since during reading we move our “attention ‘through’ both space and time” ([19], p68). For English, French, and Spanish language speakers, for example, the mental timeline is a horizontal linear line that goes in direction from left to right, with the left side representing earlier time and the right side representing later time [21]. Thus, when mentally picturing a series of events, representing earlier events on the left side would be congruent with our mental timeline as English speakers, but representing earlier events on the right side would be incongruent with our mental timeline. The left side can also represent ‘the past’ and the right side can represent ‘the future’ [22, 23]. The results of one recent study goes further to suggest that not only can we represent time using a mental timeline, but that spatial processing is required to represent time in this way. This study found that hemi-spatial neglect patients, who neglect the left side of space, also neglect the left side of the mental timeline [24]. That is, when French-speaking left hemi-spatial neglect patients were asked to learn a story about a man that described things he liked to eat in the past and things he liked to eat in the future, patients were much less likely to recall the food items he liked in the past compared to the future.

In the present investigation, we bring together the literature on space and time in language and thought, to spatial and temporal relations in long-term episodic memory and its development. Our primary goal was to test whether the direction and linearity of stimulus presentation would modulate memory for space or time. Specifically, we asked whether memory would benefit from information being presented from left to right in a linear fashion, corresponding to the mental timeline examined in previous research [13, 19].

In the present study, we operationalize memory for spatial information (space) as memory for the particular location on a computer screen in which an item was presented (e.g., “left side”), and memory for temporal information (time) as the ordinal position of the events within a temporally ordered sequence of events (temporal order; e.g., “second event”). No study to our knowledge has examined whether or how the mental timeline could influence episodic memory. Additional goals were to directly compare memory accuracy for spatial location and temporal order using the same task, and to outline the developmental trajectories of memory for time and spatial location in long-term memory since this has implications for our understanding of episodic memory development.

We can glean some insight into episodic memory development and the relation between spatial and temporal memory from the small corpus of studies that have tested both spatial and temporal memory in the same group of child and adult participants. Using a memory task involving the presentation of triplets of objects on a computer screen, Lee and colleagues found different developmental trajectories for spatial and temporal memory over short delays: memory for space (location on a computer screen) reached adult levels by 9.5 years, whereas memory for time (temporal order of object triplet) showed age-related improvement into adulthood [25]. In a study with children and adolescents, differences in the developmental trajectories for spatial and temporal memory were also found using different behavioral tasks, that were not matched for response demands [26]. In this study, the opposite pattern was observed: spatial memory (assessed via accuracy of object’s original location on computer screen), but not temporal memory (assessed via accuracy of both an object’s ordinal position and order within sequence), showed continued improvement into young adulthood. Overall, the differing developmental trajectories for spatial and temporal memory performance observed in both studies suggest that space and time may, at least in part, operate independently, at least when memory is tested immediately after encoding. However, in another study, when asked to recall temporal and spatial details from a story after a 10-minute delay, 4- to 16-year-old children showed greater accuracy for spatial details (i.e., remembering which room within the house the event from the story occurred) compared to temporal details (i.e., remembering whether the event happened before or after another event in the story). Furthermore, there was no interaction with age group [27], suggesting similar developmental trajectories in long-term memory. The small literature on the topic and the mixed results within this literature highlight the need for additional studies in which spatial and temporal memory are tested in the same group of participants, using the same tasks and with manipulations that may probe the relation between the two types of memory.

Examination of neural recruitment for spatial and temporal memory may also give us insight into relations between space and time, but this literature is also mixed. On the one hand, there is evidence that memory for space and time function similarly and recruit similar brain regions. For example, adults with lesions to the hippocampus are impaired in both spatial (location on a computer screen) and temporal memory (temporal order of objects within a sequence) to the same degree [28], suggesting a shared neural substrate for memory for both types of information. On the other hand, there is evidence that memory for time (e.g. temporal order) and space (e.g., spatial location, relative distance) involve differing brain networks [29, 30] and may recruit the hippocampus in different ways [31]. Thus, it is still not clear whether, and how, spatial and temporal features of events may influence each other in long-term memory. Moreover, these studies examining memory for spatial and temporal information are not informed by insight coming from the studies reviewed earlier on how language influences thought.

### The present study

In the present investigation, we examined memory for spatial location and temporal order using the same memory paradigm in middle to late childhood and in young adults. Participants studied triplets of objects. Within each triplet, objects were presented one at a time in a specific location on the computer screen. Each object was presented in such a way that we could experimentally manipulate linearity and direction, both factors that would be influenced by the mental timeline [20, 22]. Our primary goal was to determine whether spatial location and temporal order in memory would parallel research in linguistics [13, 14], such that the spatio-temporal mental timeline would modulate memory for temporal order more than spatial location. In addition, if the predicted effects were observed, our experimental design allowed us to test which aspects of the mental timeline (linearity or direction) were the most relevant factors driving the effects. Further, we tested for developmental continuity or discontinuity by examining whether the patterns of results were similar or different for children and adults. Overall, this work has broad implications. First, evidence of an influence of the mental timeline in long-term memory for events would add to the literature on the relation between space and time in the mind [32]. Second, evidence of different developmental trajectories for spatial and temporal memory, and/or different influences of the spatiotemporal mental timeline on memory for spatial location and temporal order would impact our understanding of the organization of spatial and temporal components of the episodic memory system [33].

## Method

### Participants

One hundred fifty children and young adults participated. Two 9-year-olds were excluded from data analysis because one child’s parent reported a neurodevelopmental disorder during participation, and one child elected to discontinue participation. One 11-year-old was excluded because during the old/new recognition task (see below) he pressed the same response key (‘new’) for all trials. In addition, one young adult was excluded because he reported not following the task directions. The final sample consisted of 37 7-year-olds (17 female; age *M* = 7.60 *SD* = .28), 34 9-year-olds (18 females; *M* = 9.55, *SD* = .30); 36 10-12-year-olds (19 female; *M* = 11.37, *SD* = .65) and 39 young adults (19 female; *M* = 21.13, *SD* = 2.23). The majority (25) of children in the 10-12-year-old group were 11 years old. From here on this group will be referred to as 11-year-olds.

Children were recruited from a lab database of families who expressed interest in research participation and received $20 for participation. Adults were recruited from a university participant pool and received course credit for their participation. Participants engaged in several tasks during the session, however only the first task completed is the focus of the present study. This research was reviewed and approved by the UC Davis Institutional Review Board before the study began. Participant consent involved written informed consent obtained from parents of child participants and from young adults. Children provided verbal assent.

### Stimuli and apparatus

**Fig 1:**
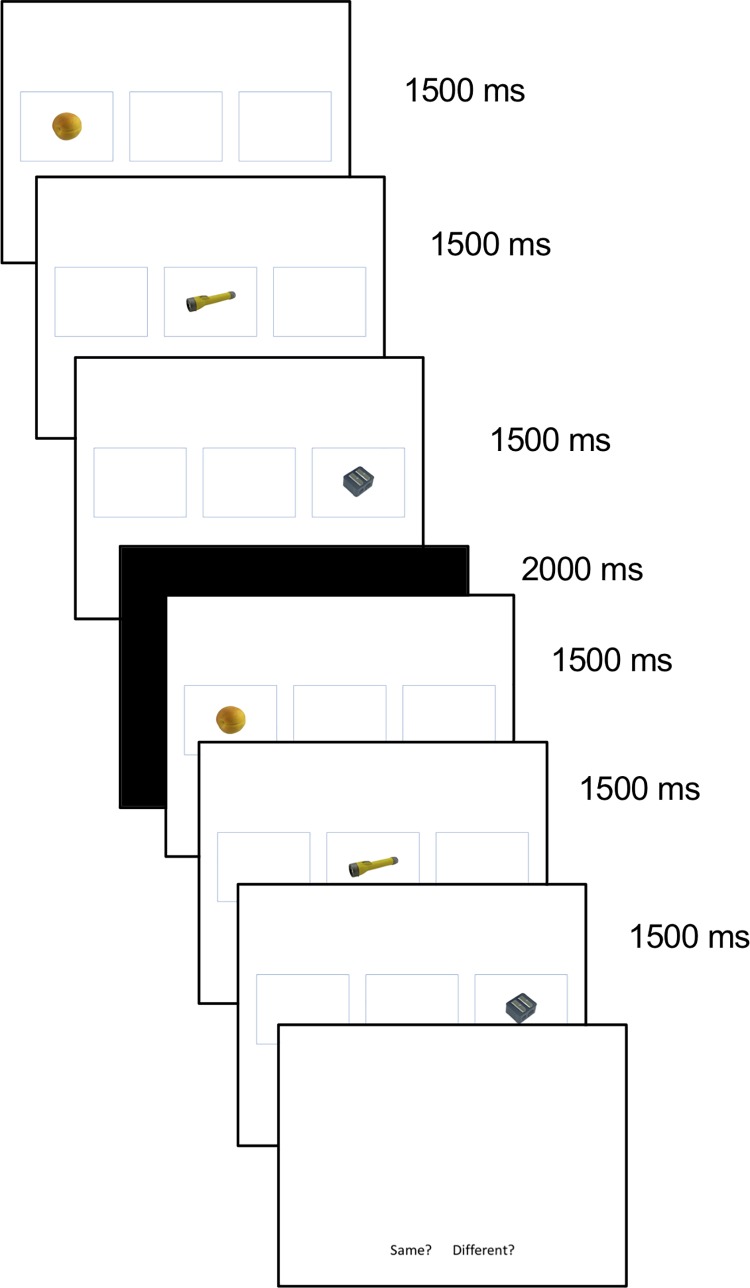
Sample encoding trial.

**Fig 2:**
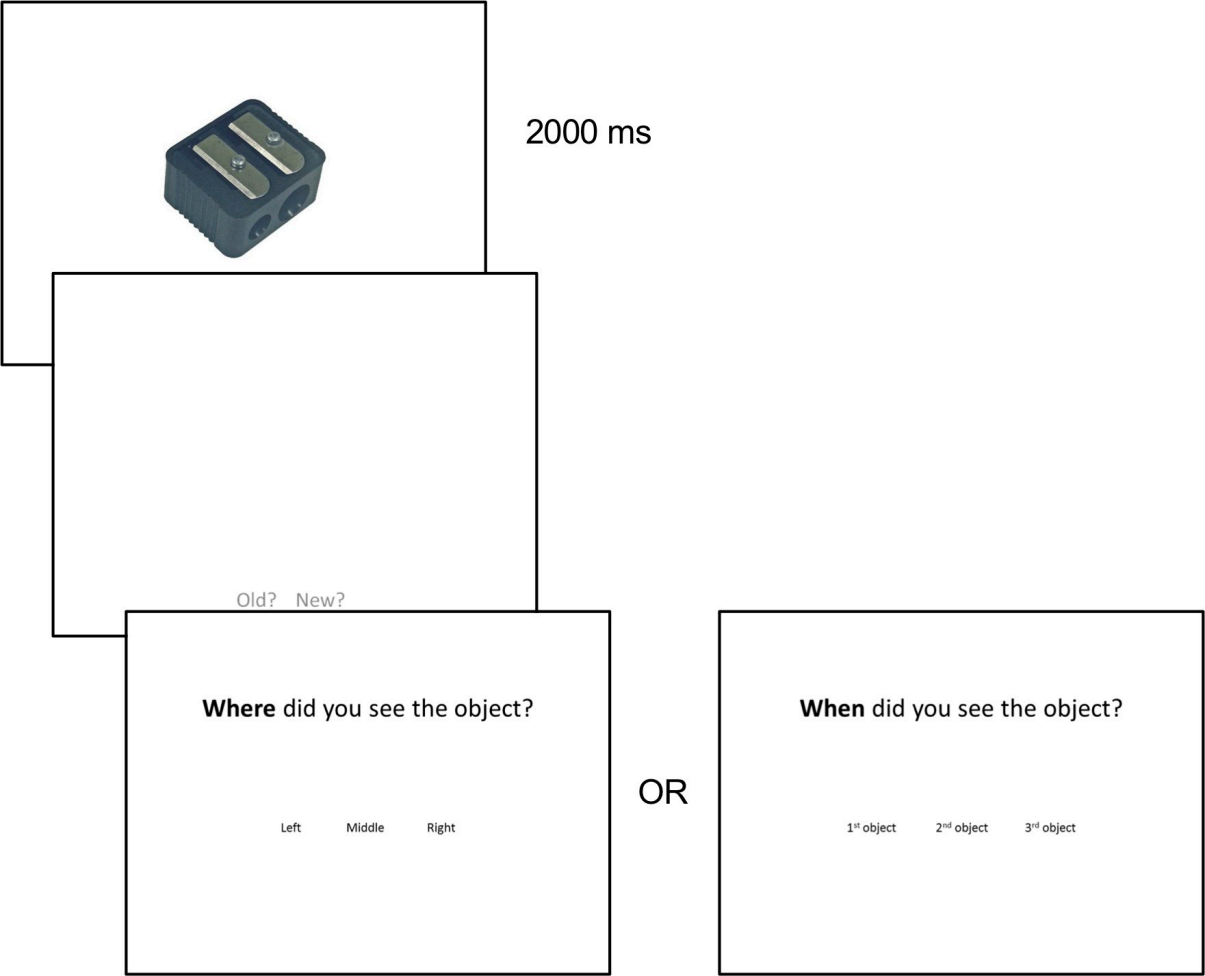
Sample retrieval trial.

Stimuli were color photographs of objects from a bank of standardized stimuli (BOSS) [34]. Stimuli were presented on a desktop computer monitor using the program DirectRT (Empirisoft Corporation) [35], and participant responses were recorded using a standard keyboard. In the encoding phase, objects were presented within rectangular boxes in either the left, middle or right position of the screen (see Fig 1), and during the retrieval phase, objects were presented at the center of the screen but larger in size so that they did not match any of the encoding displays (see Fig 2). Objects from the BOSS set were randomly assigned to form triplets presented during the encoding phase (see below). Two randomized stimulus sets were created and used across participants.

### Design and procedure

After completing IRB approved consenting procedures, children and adults participated in a computer-based memory task that consisted of study (encoding) and test (retrieval) blocks. In brief, during encoding blocks, object triplets were presented on the computer screen such that each object within the triplet had a unique spatial location (left, middle, or right box on the screen) and a unique ordinal position (1st, 2nd, or 3rd object presented) within the triplet. During the retrieval blocks, participants were presented with previously seen objects and new objects, and were asked to identify the objects seen previously (old/new recognition judgment). For each object identified as old, they were also asked to provide the spatial location or ordinal position of the object. There were 2 encoding-retrieval blocks (e.g., encoding block 1, retrieval block 1, encoding block 2, retrieval block 2). Participants were randomly selected to be either in the spatial or temporal group (i.e., they were either asked about spatial location or ordinal position during retrieval). In the final sample, there were 75 participants in the spatial group and 71 in the temporal group. We describe procedures in more detail next.

#### Description of encoding blocks

The procedure and directions for all encoding blocks were identical. Participants were always told they would be tested on their memory for objects they would see, and were told to remember where (left, middle, or right) and when (1st, 2nd or 3rd object in the sequence) the objects were presented within each triplet. In other words, participants were asked to remember both types of contextual details, even if they would subsequently be tested on only one type of detail. Given the difficulty of the task, they were told that they would see each triplet twice. To make sure that participants were paying attention, the second triplet presentation sometimes would not match the first. Participants were instructed to input on a keyboard whether the second triplet presentation was the same or different from the first. See Fig 1 for a sample encoding trial. There were 20 triplets presented for each encoding block that would later be tested (‘same’ trials). An additional 5 triplets per block were presented, but not used in the retrieval block (‘different’ trials; triplet did not match in the first and second presentation). The second presentation of the sequence in ‘different’ trials differed in both spatial location (e.g., an object was shown in a different location) and temporal order (e.g., an object was presented in a different ordinal position). Before the first block, participants were given the opportunity to practice the task using different stimuli. There was a 3-minute delay between each encoding and respective retrieval block.

#### Description of retrieval blocks

Participants were presented with old and new objects and asked to input on a keyboard whether each item was old or new. If they selected ‘old’, participants were then either asked a spatial question or a temporal question. Participants in the spatial group were instructed to select whether the object in the triplet had been shown on the left, middle or right side of the screen. Participants in the temporal group were instructed to select whether the object in the triplet had been presented 1^st^, 2^nd^, or 3^rd^. See Fig 2 for a sample trial. One object from each encoded triplet served as the test object for each retrieval trial. There were a total of 40 old trials (20 trials in each of the two blocks) and 20 new trials (10 trials in each of the two blocks; these objects were not shown during encoding).

#### Experimental manipulations

The experimental manipulation of interest was delivered during encoding. All possible combinations of the objects were represented across trials. Specifically, given that there were 3 objects in each grouping (triplet), there were 6 possible combinations of the objects within the triplet (see Table 1). We manipulated *direction* by grouping trial types that went from left to right (L-R) or right to left (R-L). We manipulated *linearity* by grouping trial types that were linear (items presented without ‘jumps’; first image of triplet presented on either left or right, second image of triplet presented in the middle, and third image of triplet presented on either right or left) or non-linear (items presented with ‘jumps’; first or last image of triplet presented in the middle). Fig 1 shows an example of a L-R Linear trial. Two of the trial groupings (LRM and MLR) contained a large jump in left to right direction, and thus can be averaged and called L-R Non-linear trials, and two of the trial types (RLM, MRL) had a large jump in right to left direction, and thus can be averaged and called R-L Non-linear trials. There were an equal number of trials for each of the four groupings presented in Table 1 (L-R Linear, R-L Linear, L-R Non-linear, R-L Non-linear).

**Table 1:** Experimental manipulation: The six possible trial types presented during encoding, and how they are grouped into four conditions.

		Linearity	
		Linear	Non-linear
Direction	Left to Right (L-R)	LMR	LRM, MLR
	Right to Left (R-L)	RML	RLM, MRL

*Note*. L = left, M = middle, and R = right, going in order from left to right space. There were 10 trials total in each of the four conditions.

This grouping of trial types allows us to test specific predictions about memory accuracy during the retrieval phase. We could test whether our experimental manipulation influenced accuracy scores, and if they did, the grouping of the trial types allows us to test the importance of linearity and the importance of direction, both characteristics of the spatiotemporal mental timeline. If, for example, linearity without direction alone was driving the effects, then we would expect no differences between L-R Linear and R-L Linear trial types, however accuracy for both of these trial types would be higher than both Non-linear trial types. On the other hand, if both linearity and direction were driving the effects, then we would expect that the L-R Linear trials to have the highest accuracy scores and differ from all other types, and we would expect R-L Non-linear trials to show the lowest levels of accuracy. As a reminder, L-R Linear trials are most congruent with the mental timeline for English speakers; R-L Non-linear trials are least congruent with the mental timeline.

## Results

### Encoding phase

Accuracy for the same-different encoding task was high for all age groups. For the spatial test group, the average (and standard deviation) percentage accuracy across the two blocks for 7-year-olds, 9-year-olds, 11-year-olds, and young adults, were 91.30 (.07), 92.23 (.12), 96.42 (.04), and 98.42 (.02), respectively. For the temporal test group, the average (and standard deviation) accuracy for 7-year-olds, 9-year-olds, 11-year-olds, and young adults, were 95.06 (.05), 95.76 (.06), 94.82 (.05), and 97.20 (.03), respectively. In order to determine whether there were age-related differences, or differences in encoding accuracy between the context test groups, we conducted an Age Group (7-year-olds, 9-year-olds, 11-year-olds, young adults) X Test Group (spatial, temporal) Analysis of Variance (ANOVA). We found a main effect of Age Group, *F*(3, 146) = 4.09, *p* < .01, *η*_p_^2^ = .08. Pairwise comparisons, with Bonferroni correction, revealed that 7-year-olds were less accurate than young adults (*p* < .01). There was a marginal effect between the 9-year-olds and young adults (*p* = .06), and all other age group comparisons were not significant (*p*s > .57). Please note that all following pairwise comparisons were conducted with Bonferroni correction. Importantly, there was no main effect of Test Group (*p* = .28), and no interaction between Age Group and Test Group (*p* = .11). Thus, encoding task performance did not differ between the participants that were assigned to later receive either a spatial or temporal context test question. Although encoding accuracy was very high overall, since there were some age-related differences we only examined correct encoding trials for the retrieval phase analysis.

### Retrieval phase

#### Old-new recognition

We conducted a Test Group (spatial, temporal) x Age Group ANOVA on proportion of hits, and found a main effect of Age Group, *F*(3, 146) = 3.24, *p* < .05, *η*_p_^2^ = .07. Pairwise comparisons with Bonferroni correction, revealed that 7-year-olds had fewer hits than 11-year-olds (*p* < .05); there were no other differences between age groups. Importantly, there was no main effect of Test Group (*p* = .39), nor an interaction between Test Group and Age Group (*p* = .63). Thus, recognition memory performance did not differ between the participants that were assigned to receive either a spatial or temporal context test question. For the parallel analysis with false alarms, there were no main effects of Age Group, no main effects of Test Group or interactions (all *p*s > .16). Values for hits and false alarms are provided in Table 2.

**Table 2:** Old-new recognition memory means (and standard deviations).

	7-year-olds	9-year-olds	11-year-olds	Young Adults
Hits	.80 (.15)	.87 (.11)	.88 (.11)	.87 (.11)
False Alarms	.10 (.16)	.07 (.13)	.05 (.06)	.07 (.06)

#### Spatial versus temporal memory

Proportions of correct responses for the context test question (spatial *or* temporal) were calculated from all trials that were hits (i.e., correctly recognized as old during recognition). We conducted a 4 (Age Group: 7-, 9-, 11-year-olds, young adults) x 2 (Test: spatial vs. temporal) x 2 (Linearity: linear vs. non-linear) x 2 (Direction: L-R vs. R-L) ANOVA. There was a main effect of Age Group, *F*(3, 137) = 3.96, *p* < .01, *η*_p_^2^ = .08. Pairwise comparisons, with Bonferroni correction, revealed that overall seven-year-olds were less accurate than 11-year-olds (*p* < .05) and young adults (*p* = .07). There was a main effect of Test, *F*(1, 137) = 66.17, *p* < .0001, *η*_p_^2^ = .33. Overall, participants performed better in the spatial compared to the temporal test. There was also a main effect of Linearity, *F*(1, 137) = 43.03, *p* < .0001, *η*_p_^2^ = .24, a main effect of Direction, *F*(1, 137) = 27.32, *p* < .0001, *η*_p_^2^ = .17, and Test x Linearity, *F*(1, 137) = 57.09, *p* < .0001, *η*_p_^2^ = .29, and Test x Direction, *F*(1, 137) = 17.14, *p* < .0001, *η*_p_^2^ = .11, interactions. These were subsumed by a Test x Linearity x Direction interaction, *F*(1, 137) = 4.72, *p* < .05, *η*_p_^2^ = .03. There was also a marginal Age Group x Linearity interaction, *F*(3, 137) = 2.65, *p* = .05, *η*_p_^2^ = .06. There were no other interaction effects with Age Group and any of the other variables *(F*s < 2.10, *p*s > .10).

**Fig 3:**
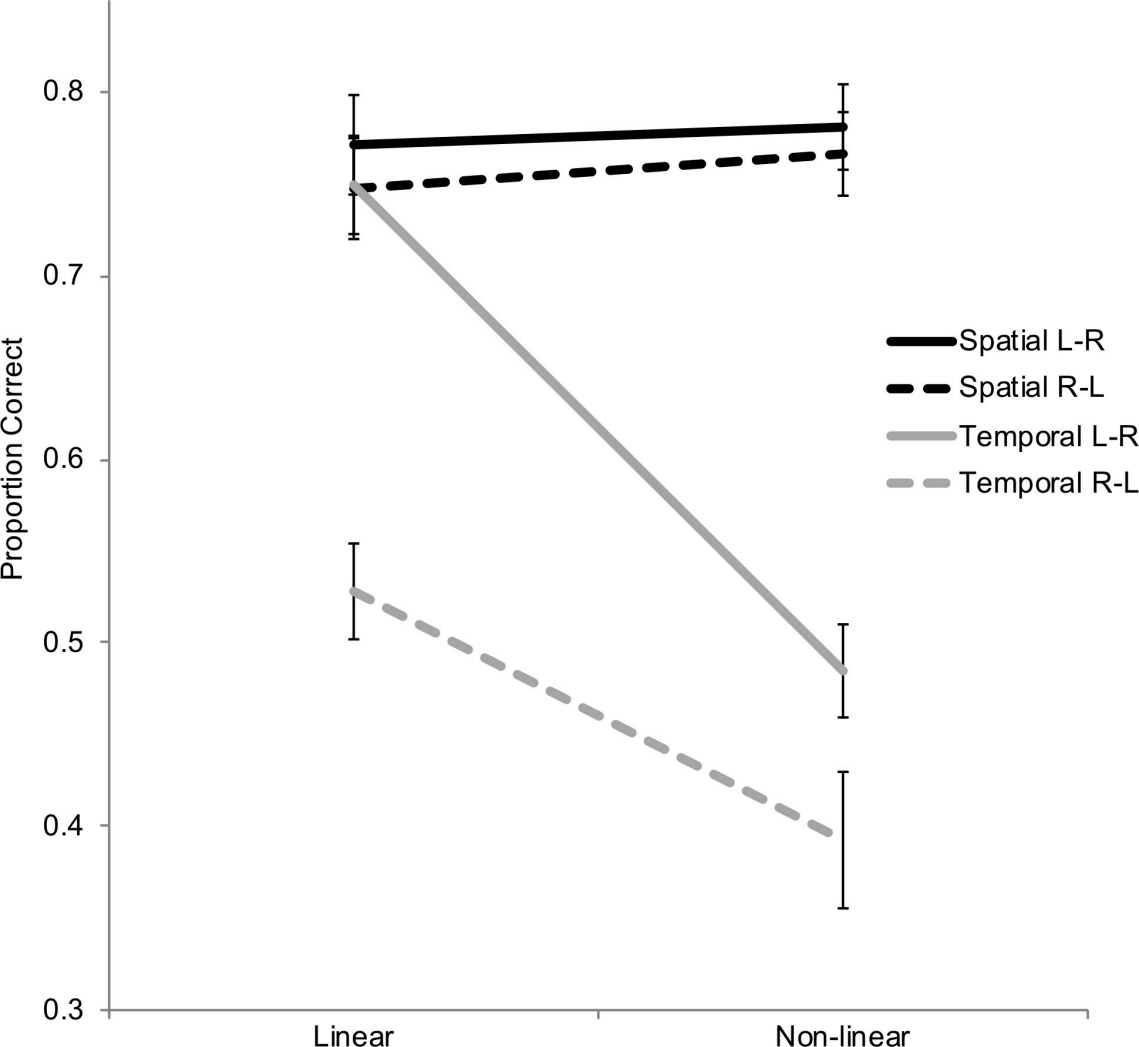
Context test question means for spatial test group (black lines) and temporal test group (gray lines). Error bars are +/- standard error.

To follow-up the Test x Linearity x Direction interaction, we conducted an ANOVA for each test group separately (across age groups). For the spatial test (Fig 3, black lines), we found no main effects of Linearity or Direction, nor a Linearity x Direction interaction (*p*s > .31). However, for the temporal test (Fig 3, gray lines), there was a main effect of Linearity, *F*(1, 70) = 75.85, *p* < .0001, *η*_p_^2^ = .52, a main effect of Direction, *F*(1, 70) = 32.84, *p* < .0001, *η*_p_^2^ = .32, and a Linearity x Direction interaction, *F*(1, 70) = 8.75, *p* < .005, *η*_p_^2^ = .11. Pairwise comparisons with Bonferroni correction adjustments showed accuracy was higher for the Linear L-R trial type compared to all other trial types (*p*s < .0001); Accuracy did not differ between Linear R-L and Non-linear L-R trial types (*p* = .69); Accuracy was lowest for the Non-linear R-L trial type: Non-linear R-L was lower than both Linear R-L and Linear L-R (*p*s < .0005); the difference between Non-linear R-L and Non-linear L-R trial types did not reach conventional levels of significance (*p* = .08). Note that accuracy for the temporal context test question within the Non-linear R-L condition was not above chance level responding, *t*(70) = 1.68, *p* = .10; Accuracy for all other trial types was above chance responding (*p*s < .0001).

We explored the marginal Age Group x Linearity interaction, and found that (across Test and Direction) for 7-year-olds there was no difference between Linear and Non-linear trials, *t*(36) = .43, *p* = .67, but for all other age groups Linear was higher than Non-linear (*t*s > 3.25, *p*s < .005). Note that we conducted this analysis to be thorough, however, we are cautious to make strong claims about this finding given that the analysis is across Test.

Accuracy scores for each age group separately are presented in S1 Fig and S2 Fig.

## Discussion

In the present study, we examined memory for events along with their temporal and spatial contexts in children and adults. Our goal was to examine developmental differences in memory for spatial location and temporal order, two central aspects of episodic memory content. Critically, we examined these aspects of episodic memory by manipulating how arbitrary event sequences were presented during learning. Previous work in domains outside of memory revealed that temporal judgments are influenced by spatial judgments more than vice versa [16], and the mental timeline supports representations of time [32]. Here, we tested whether long-term memory would be influenced by factors relevant to the mental timeline, linearity and direction. Objects were presented such that spatial location and temporal order overlapped in a linear fashion or did not, and either in the left to right or right to left direction.

Our major finding was that when events were presented during encoding linearly, from a left to right direction, their temporal order was remembered best. This is consistent with the idea that children and adults alike use a mental timeline, in which spatial and temporal dimensions coincide, to guide their successful retrieval of memory for temporal order. Given that the mental timeline relies on spatial distribution of objects, it could have been expected some effects on memory for spatial location. Instead, whether or not items were encoded consistent with participants’ mental timeline did not seem to affect accuracy in memory for spatial locations. The failure of effects for spatial memory suggests that spatial features can be successfully retained independent of changes occurring in other contextual features surrounding the event. On the other hand, temporal memory was influenced by how well the items were encoded according to the mental timeline. Interestingly, only retrieval of the individual item’s temporal order was necessary during test; this was not a task that required memory for temporal order of all items in the sequence. Yet the linearity and direction (spatial properties) of all three items in the encoded sequence influenced how well the temporal order of the individual item within the sequence would be remembered.

In addition to showing that spatial context, based on the mental timeline, affects temporal memory, our experimental conditions allowed us to examine how directionality alone, which capture spatial information, affected temporal memory. Accuracy was highest in the Linear L-R condition, which most matches the left-to-right mental timeline, and accuracy was lowest in the Non-linear R-L condition, which least matches the left-to-right mental timeline. Accuracy did not differ between the Linear R-L condition and the Non-linear L-R trial type, which suggests that both linearity and direction are important factors.

Our study adds to the corpus of investigations showing that temporal information is represented by and/or influenced by a mental timeline; it adds to the work showing that temporal information can be spatially organized [36–38]. For example, both English-speaking children and adults physically place items representing earlier time (e.g., breakfast) to the left and later time (e.g., dinner) to the right [36]. We found that when earlier items in a sequence were presented on the left, and later items within the same sequence were presented on the right, children and adults remembered the temporal order of the items better. Thus space, via the mental timeline, influences how the mind *represents*, and based on the results of the present study, how the mind *remembers*, time. However, there seem to be limits to the power of this mental timeline. For example, as discussed by Casasanto and Bottini [39], it is common to use spatial metaphors to describe time or temporal structure (e.g., February is closer than March), but not common to talk about spatial metaphors that directly map onto the mental timeline. For example, it is unheard of to state that February comes to the left of March. Similarly, in memory, we directly manipulated the presentation of items to test the influence of the mental timeline. However, we do not claim that all events are encoded and remembered based on a left-to-right mental timeline. Future studies should examine the span and limits to the influence of the mental timeline on long-term episodic memory. Still, it may be possible to capitalize on the effects described in the present study to determine whether the mental timeline or spatial organization can be used to aid in memory retention. Older adults seem to have more difficulty retaining temporal information compared to spatial information [40], thus it may be possible to exploit the mental timeline (and the relatively spared spatial memory) to remember temporal information. To our knowledge, no such mnemonic strategy exists, although spatial memory has long-been exploited to remember lists of items and their sequential order using the method of loci [41, 42].

Age-related differences, as expected, were found in both item recognition and memory for contextual information. Overall, the most pronounced age-related change seemed to be between the 7-year-old and 11-year-old age groups, which is consistent with what Guillery-Girard and colleagues [26] noted as a “peculiar period in late childhood…crucial for the developmental time course of episodic memory” (p. 1). In the present research, 11-year-old accuracy was, for the most part, adult-like. This trajectory in which there seems to be a significant change between age 7 and 10 or 11 is consistent with behavioral studies that have tested memory for events and their context [7, 8, 43, 44]. (There seems to also be a period of change in early childhood [45].) For example, in a task that required memory for object locations, 7-year-olds made significantly more errors than 9-year-olds, 11-year-olds and adults [43]. In a computer task that required memory for the order of object sequences, 7-year-olds were less accurate than both 10-year-olds and young adults [8]. The present study findings are also consistent with evidence showing age-related changes in the hippocampus in late childhood [46–49], a brain region that supports binding of events and their temporal and spatial contexts [50].

We did not find an interaction between context and age group. One could expect that there would have been more age-related improvements in temporal memory compared to spatial memory based on previous research showing that temporal memory for separate episodes exhibits continued age-related improvement into late childhood [8, 51–54], whereas there is evidence of relative competence in spatial memory in early childhood [55, 56]. Differing trajectories for spatial and temporal memory were also found in two studies using short-term memory tasks [25, 26]. Our finding of a distinct, but parallel trajectory for spatial and temporal memory is in line with the only other study that has examined both contexts in a long-term memory task: Picard and colleagues [27] also found that spatial memory accuracy was higher than temporal memory across all age groups. Future work, in which a wide range of age groups are tested using both short-term and long-term memory tasks are needed to better document the developmental trajectories for memory for events and their spatial and temporal contexts.

Future research should also examine the impact of verbal encoding of the stimuli. In the present research, the individual objects and their spatial (left, middle or right) and temporal (1^st^, 2^nd^, or 3^rd^) features within the event sequences were nameable. That is, participants could verbally rehearse the sequences during the encoding phase for both spatial and temporal memory. However, we may expect different patterns if both the objects and the contextual information about the stimuli were less likely to be verbally rehearsed. When stimuli are not objects that can be easily named and thus less likely to be verbally coded for item memory or spatial and temporal features, adults do not show a difference in accuracy for spatial and temporal memory, though children do [25, 50, 57]. In the present study, adults showed greater accuracy for memory for an object’s location on a computer screen, compared to memory for an object’s temporal order within a sequence, which could mean that verbal codes for space (binding nameable object with verbal spatial feature; “shoe, left”) are better retained than verbal codes for time (binding nameable object with verbal temporal feature; “shoe, 2^nd^”).

Differences in the ability to verbally code spatial and temporal information may explain why the findings of the present research seem inconsistent with the findings from Rondina and colleagues [33] who examined the relations between temporal and spatial features using a short-term memory paradigm. Adults were presented with triplets of non-nameable objects at different locations on a computer screen and after a 2 second delay, were presented with the triplets such that the spatial and temporal relations between the objects were the same (i.e., same spatial configuration between the objects, and same temporal order), or were manipulated (i.e., spatial relations were manipulated, temporal relations were manipulated, or both the spatial and temporal relations were manipulated). Although the objects were not nameable, given the very short delay, authors state that temporal features could be coded verbally (“1^st^, 2^nd^, 3^rd^”), whereas the spatial features could not. Researchers found that overall accuracy was higher for the temporal than the spatial information. Moreover, they found that manipulating temporal relations affected participants’ spatial memory accuracy, but manipulating spatial relations did not affect participants’ temporal memory accuracy. This finding suggests an *asymmetric* relation between space and time in the opposite direction to that predicted by the language literature [13]. The findings are also contrary to the present research since we found that accuracy was higher for spatial location than temporal order. (There is another short-term memory study suggesting independence of space and time [58].) Further research is needed to examine whether there is a switch in the hierarchical relation between space and time from short-term to long-term memory, or whether the differing results are due to differences in how spatial and temporal information is coded in memory. Adapting the elegant study by Rondina and colleagues [33] to incorporate a longer delay would be very interesting since it could allow for a test of whether memory for space influences memory for time, whether memory for time influences memory for space, or whether memory for space and time appear to function independently in long-term memory. Future work could also examine whether participants are more likely to accurately recall space if they also accurately recall time, or vice versa, and whether this changes across development.

There is evidence that the mental timeline is influenced by language, and so it would be informative if we conducted the present study in cross-cultural samples. Although English speakers represent time in a horizontal mental timeline, there is evidence that Mandarin speakers can represent time in a vertical mental timeline also [21]. Thus, it is possible that native Mandarin speakers would show attenuated levels of the memory effects reported here. If so, this would add to the corpus of evidence on the asymmetric relation between time and space in the mind [15], and also be a demonstration of the impact of language experience on memory. In terms of the development of the mental timeline, our results suggest that by 7 years of age, children have reached a reading level that allows them to make use of the mental timeline. This is consistent with studies that have examined the emergence of the mental timeline [59, 60]. For example, Droit-Volet and Coull [59] found that the mental timeline was not evident at age 5, but was evident at age 8 and 10 years. Our results show that not only are 7-year-olds able to form a mental timeline, but that they can exploit it in similar ways to older children and adults in order to support memory.

Memory for spatial and temporal features of events are integral to theories of episodic memory [61–63]. The importance of binding together an event with its spatial and temporal context is also necessary for our ability to form life stories and highlighted in theories of autobiographical memory [64]. The present research suggests that there may be an asymmetric organization within our memories such that spatial features (spatial location) can be better retained than temporal features (temporal order) in long-term memory, and that encoding of temporal order can be supported by a spatiotemporal mental timeline. Additional research in this area is warranted. For example, future work will need to examine the impact of using a response pad that is spatial in nature (e.g., layout matches alignment of a horizontal mental timeline). Weger and Pratt [65] found that young adults were faster at categorizing images of actors popular in the distant past (e.g., Charlie Chaplin) with their left index finger, compared to their right index finger, and that they were faster at categorizing images of actors popular more recently (e.g., Brad Pitt) with their right finger, compared to their left index finger. In the present work, the way the temporal question was presented during retrieval and the required layout of the fingers used to respond could have influenced retrieval in a way we could not detect (see S1 Analyses). Thus, follow-up studies can test the limits of the present study findings. However, together this work underscores some important dimensions, linearity and directionality, uniquely supporting memory for temporal order.

## Supporting information

S1 Analyses Post-hoc supplemental analyses. (PDF)

S1 Fig Spatial test mean accuracy for each age separately. Error bars are +/- standard error.
(TIFF)

S2 Fig Temporal test mean accuracy for each age separately. Error bars are +/- standard error.
(TIFF)
