# Supplementary material for: Space and time in episodic memory: Effects of linearity and directionality on memory for spatial location and temporal order in children and adults
Source: PLoS One. 2018 Nov 8;13(11):e0206999. doi: 10.1371/journal.pone.0206999 (PMC6224083; doi:10.1371/journal.pone.0206999)
Supplement: S1 Analyses — (PDF) [file pone.0206999.s001.pdf]

Supplemental Analyses

In Figure 2, the question asked to the participant shows 1st/2nd/3rd appearing from left to right on the screen. To check whether this caused an inherent bias for participants to use a left to right mental timeline to represent temporal order we conducted the following post-hoc analyses. We thank an anonymous Reviewer for suggesting we address this possibility.

It not possible for the screen to introduce a bias to learning during Block 1, since this screen was not available during encoding or delay, and only first presented in the retrieval phase of Block 1. However, it is possible that this introduced a bias for Block 2 for the temporal group. To check, we conducted three types of analyses comparing Block 1 and Block 2. If this screen introduced a bias in how participants learned the stimuli in the temporal group, then we would expect to see a different pattern in results for Block 2. First, we conducted paired t-tests comparing Block 1 and Block 2 temporal question accuracy for each of the 4 trial types. We found that there were no significant differences in accuracy for each trial type (ps > .05, ts < 1.9). Second, we conducted a Linearity (linear, non-linear) x Direction (L-R vs. R-L) x Block (Block 1 vs. Block 2) repeated measures (RM) ANOVA for the temporal group. We found main effects of linearity, direction and a linearity x direction interaction, as expected. Importantly, there were no interactions with Block. Specifically, there was no Linearity x Block interaction ($F(1, 53)=.75$, $p=.30$), no Direction x Block interaction ($F(1, 53)=.17$, $p=.69$), nor a Linearity x Direction x Block interaction ($F(1, 53)=.50$, $p=.48$). There was only an expected Main effect of Block ($F(1, 53)=7.4$, $p=.009$), such that accuracy across trial types was higher in Block 2 compared to Block 1. As a final check, we conducted a Linearity x Direction RM ANOVA for only Block 1. We are cautious when interpreting this analysis, given that it is based fewer trials than in the full study. Nevertheless, we still found effects of linearity ($F(1, 61)=50.9$, $p<.0001$;

linear greater than non-linear) and direction ($F(1, 61)=20.6$, $p<.0001$; L-R greater than R-L). Thus, these patterns match those reported in the manuscript when we averaged across Block 1 and 2. Given these analyses, we do not believe that the screen shown in Figure 2 affects our claims.
