# Supplementary figures and images for: Space and time in episodic memory: Effects of linearity and directionality on memory for spatial location and temporal order in children and adults
Source: PLoS One. 2018 Nov 8;13(11):e0206999. doi: 10.1371/journal.pone.0206999 (PMC6224083; doi:10.1371/journal.pone.0206999)

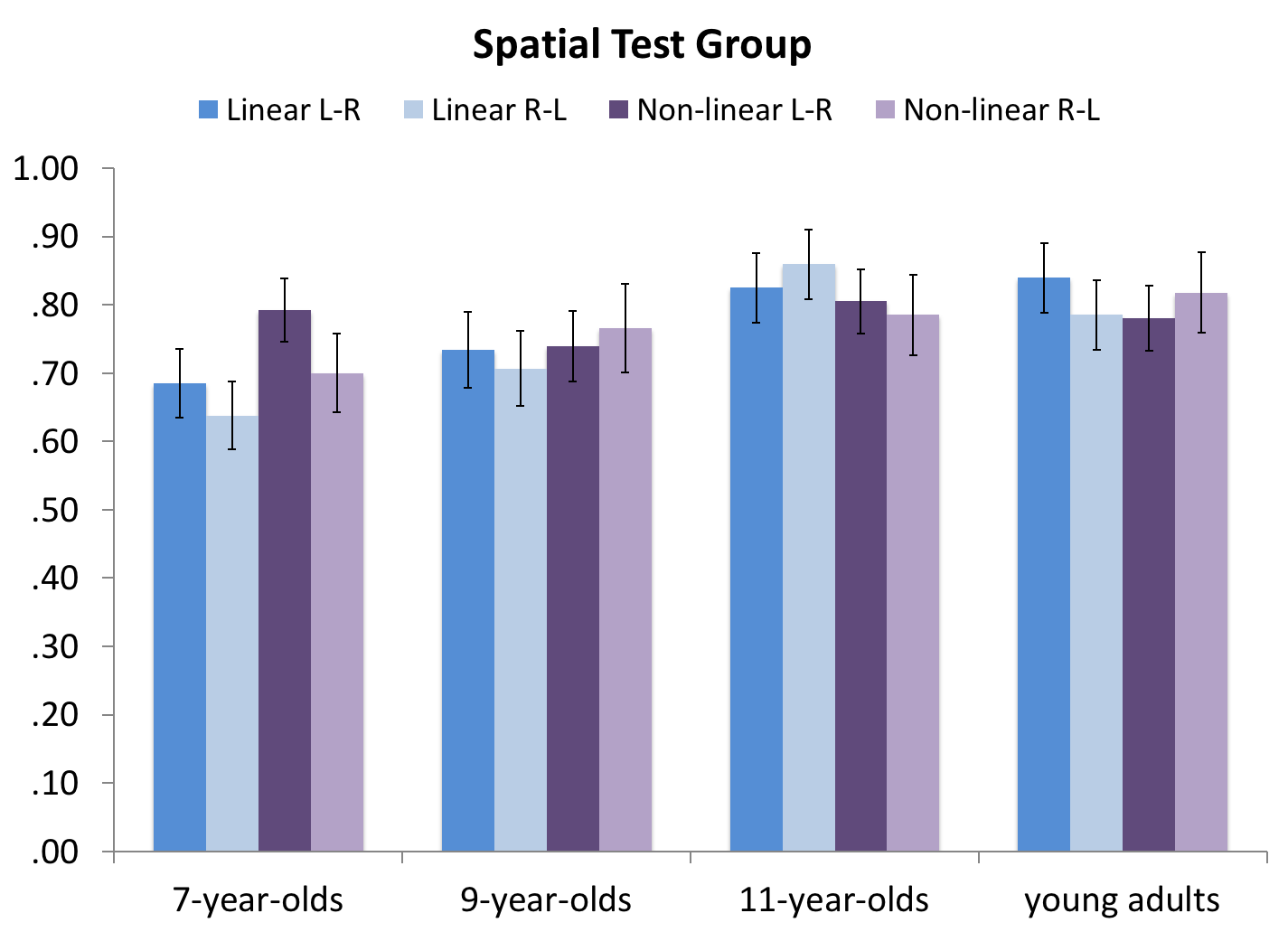

Supplement: S1 Fig — Error bars are +/- standard error. (TIFF) [file pone.0206999.s002.tiff]

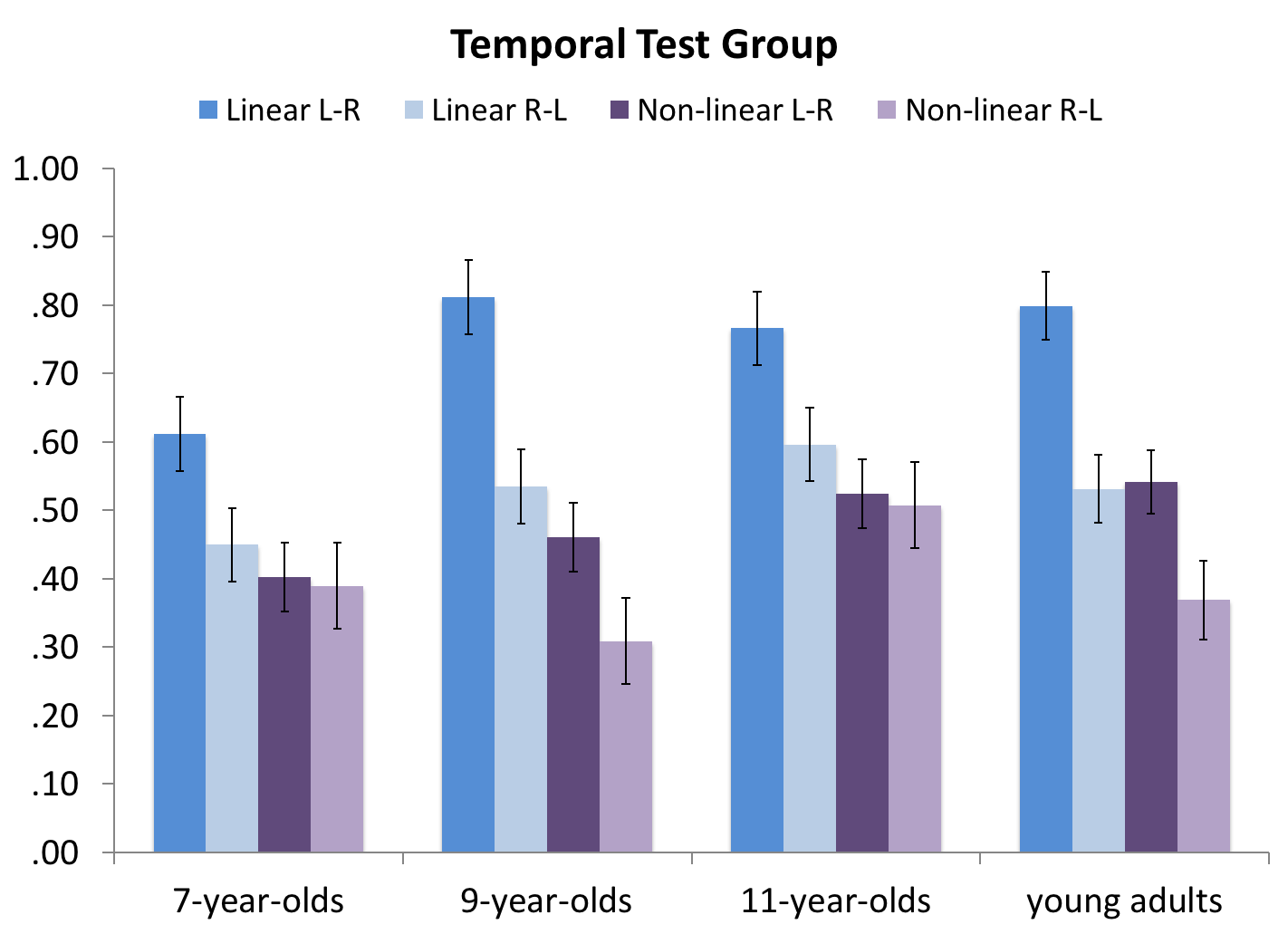

Supplement: S2 Fig — Error bars are +/- standard error. (TIFF) [file pone.0206999.s003.tiff]
